# Treatment Fidelity in a Feasibility Trial of the Aphasia Intervention, Virtual Elaborated Semantic Feature Analysis

**DOI:** 10.1111/1460-6984.70054

**Published:** 2025-05-23

**Authors:** Niamh Devane, Sofia Mazzoleni, Nicholas Behn, Jane Marshall, Stephanie Wilson, Katerina Hilari

**Affiliations:** ^1^ Department of Language and Communication Science City St George's, University of London London UK; ^2^ East London NHS Foundation Trust London UK; ^3^ Centre for Human‐Computer Interaction Design City St George's, University of London London UK

**Keywords:** aphasia, treatment, validity

## Abstract

**Background and Aims:**

The reliability and validity of an intervention can be improved by checking treatment fidelity (TF). TF methods identify core components of an intervention, check their presence (or absence) and identify threats to fidelity. The Virtual Elaborated Semantic Feature Analysis (VESFA) intervention comprised individual sessions of word‐finding treatment and group sessions of conversation practice. All sessions were delivered in the virtual world of EVA Park. This paper describes the TF in the VESFA trial that explored (1) if the treatment was delivered as planned, (2) which components influenced treatment adherence scores and (3) the reliability of the fidelity checklists.

**Methods and Procedures:**

Strategies to improve fidelity were employed in the study design, the delivery of treatment, treatment receipt and treatment enactment. Two fidelity checklists were developed with input from advisors with aphasia to establish the core components of the intervention (individual and group). During the trial, treatment sessions were video‐recorded. A sample of 20% of sessions was randomly selected for adherence rating. Seven research students were trained to rate the videos using the fidelity checklists. Inter‐ and intra‐rater reliability was established.

**Outcomes and Results:**

Study design strategies ensured 94% of sessions ran as planned and 75% of participants (12/16) received over 90% (>36/40h) of the intended dose. The average TF across all sessions rated was 81%, demonstrating a high degree of fidelity in the delivery of the VESFA intervention. The fidelity of the individual sessions was lower (78%) than the group elements (84%). The components that most threatened treatment adherence were (1) providing a rationale for the activities and (2) specific feedback for performance. Nevertheless, participants consistently practised target words both in individual sessions and in conversations in the group sessions, demonstrating treatment receipt. Ninety‐four percent of participants (14/15) reported the words and phrases practiced in EVA Park were used in real‐world conversations, indicating treatment enactment. The fidelity checklists were reliable: Inter‐rater reliability was moderate (average Kappa of 0.76) and intra‐rater reliability was strong (average Kappa of 0.89).

**Conclusions and Implications:**

A range of TF strategies were embedded within the trial protocol leading to high adherence to the core components of the VESFA intervention. Findings add to the evidence that aphasia therapies can be administered faithfully within the virtual environment of EVA Park.

**Trial Registration:**

The feasibility trial was not registered.

**WHAT THIS PAPER ADDS:**

*What is already known on this subject*

- Monitoring treatment fidelity improves both internal and external validity. Reports of treatment fidelity from aphasia trials are increasing, but the guidance is not yet applied uniformly.

*What this study add to the existing knowledge*

- This study demonstrates how treatment fidelity guidance has been applied across a range of fidelity areas to monitor and support a feasibility trial of a novel aphasia intervention. It is a rare reporting of strategies to monitor treatment enactment.

*What are the potential or actual clinical implications of this study?*

- This study adds to the evidence base for the VESFA intervention, demonstrating that the intervention can be delivered faithfully to the manual. It builds on the evidence base for treatment fidelity monitoring in aphasia, broadening the strategies to improve the validity of interventions.

## 1 Introduction

Treatment fidelity (TF) is defined as the methods used ‘to monitor and enhance the reliability and validity of behavioural interventions’ (Bellg et al. 2004, 443). Behavioural interventions are developed based on stakeholder views, underlying theory and programme logic (O'Cathain et al. 2019). Then they are tested. Confident conclusions about the outcomes cannot be drawn unless we know that the intervention was delivered as intended. Thus, well‐designed TF methods improve internal validity. Monitoring TF also leads researchers to identify issues in the delivery of the intervention, such as components with poor adherence. This may lead to refinements of the treatment protocol or to the methods used for training intervention providers. TF monitoring can enable hypotheses to be developed about which core components drive change that can be attributed to treatment, not just any change. This supports the replication of the intervention, and enhances external validity (Borrelli 2011).

### 1.1 Treatment Fidelity Guidance

There are following five areas where guidance exists to enhance TF: (1) the design of the study, (2) the training of intervention providers, (3) the delivery of treatment, (4) the receipt of treatment and (5) the enactment of treatment skills (Bellg et al. 2004). Taking each in turn, the *study design* can ensure that the treatment is in line with the underlying theory and that equal doses are planned within each condition. *Training intervention providers* aim to ensure the participants receive the same intervention. The *delivery of treatment* monitors that the delivery is adhering to the treatment protocol. This is often assessed by watching intervention sessions and rating them against a fidelity checklist of core components. The advice on the percentage of sessions to be rated is 10% minimum but optimally 20% (Behn et al. 2023), indeed, most studies report 10%–20% (Hinckley and Douglas 2013). Direct observation of sessions using a priori coding categories is considered the gold standard (Brogan et al. 2019). The *receipt of treatment* checks that participants can perform the targeted skill following treatment. This is most often achieved through the administration of relevant outcome measures before and after an intervention. Receipt of treatment is also addressed by the skill of the provider (e.g., providers of aphasia treatment are trained to facilitate communication), by inclusion/exclusion criteria which ensure that participants can access the treatment and by ensuring learning is incremental to support achieving the relevant skills (Behn et al. 2023). Finally, the *enactment of treated skills* monitors that participants can perform the targeted skill following the treatment in real‐world settings. Difficult to assess, this aspect can be explored through post‐therapy interviews, for example, with treatment participants and/or their family members. A good example comes from the Big Cactus study where participants were interviewed about factors that were associated with adherence (Harrison 2019) and videos analysed the use of the target words in conversation. It has been argued that following the Bellg guidance (Bellg et al. 2004), outlined above, increases TF and, thereby improves ‘the power to detect effects that might otherwise have been obscured by variance’ (Spell et al. 2020, p288).

### 1.2 Treatment Fidelity in Aphasia

Published accounts of TF in aphasia interventions are increasing. A review of TF reporting published in 2013 identified only 21 studies in the previous 10 years that had published TF (Hinckley and Douglas 2013). A later review found 37 studies in the following 5 years (2012–2017), however, only one article in this review contained all five elements of TF (Brogan et al. 2019). Most recently, the Trials for Aphasia Panel of the Collaboration of Aphasia Trialists developed guidelines for developing, monitoring and reporting fidelity in aphasia trials. They supplemented the Brogan review and explored seven RCTs that were either completed or ongoing in the years 2017–2021 (Behn et al. 2023). All five areas of fidelity were addressed to varying degrees in four RCTs. The least reported element in this review was treatment enactment. TF, key to our understanding of whether a treatment is valid, is now more consistently reported in aphasia trials.

This paper reports on the TF of a complex speech and language therapy intervention, Virtual Elaborated Semantic Feature Analysis (VESFA; Devane et al. 2025) during a feasibility randomised controlled trial. The VESFA intervention combined individual sessions of elaborated semantic feature analysis (Efstratiadou et al. 2019) (4 × 60‐min sessions per week) with group conversations (2 × 90‐min sessions per week) situated in the virtual world EVA Park in an 8‐week, 40h programme.

TF data for interventions delivered in EVA Park were reported in three previous studies as follows: a single word, social support and script interventions (Marshall et al. 2018; Marshall et al. 2020; Marshall et al. 2023). In these studies, fidelity checking addressed study design, treatment delivery and treatment receipt. In terms of treatment delivery, between 20% and 32% of treatment sessions were rated for the delivery of core components. Adherence ratings showed that 72.7%–92% of core components were present in the sessions. Inter‐rater reliability was *substantial* (Kappa: 0.63) to *almost perfect* (Kappa: 0.92) (Landis and Koch 1977; Marshall et al. 2020; Marshall et al. 2023). Where reported, EVA Park studies have shown high adherence to core treatment components.

There are few reports of TF monitoring in previous studies of SFA (Evans et al. 2021; Gravier et al. 2018; Kendall et al. 2019; Kladouchou et al. 2017). SFA is highly prescribed making monitoring TF relatively straightforward. Indeed, where TF has been reported for SFA, adherence to the protocol was above 95% (Evans et al. 2021; Kendall et al. 2019). Elaborated SFA (ESFA) is more complex but adherence to the protocol in a previous study was high, with a treatment adherence rating of >90% (Kladouchou et al. 2017).

Identifying the core components of SFA has been explored (Boyle 2010; Evans et al. 2021; Gravier et al. 2018; Quique et al. 2019; Sze et al. 2020). In a typical SFA protocol, participants identify the features of a target word using an SFA chart as follows: category, use, action, properties, location and association (Boyle 2004). Feature generation, not feature repetition, appears to be a key driver of change in SFA studies (Boyle 2010; Evans et al. 2021; Gravier et al. 2018). High dose (e.g., 15 sessions) has a positive impact on outcomes, for both treated and untreated items (Quique et al. 2019). Recently, a study explored all variables that might influence word‐finding outcomes (Sze et al. 2020). Providing the written form of the target as a cue was found to be a good predictor of outcomes, as was the provision of cues, dose parameters (number of sessions, number of times items were named) and the provision of feedback (Sze et al. 2020). This literature informed the development of the ESFA sessions and TF checklist within the VESFA intervention.

The core components of conversation groups are less well‐researched. One study proposes the mechanisms of change that support improved well‐being within community aphasia groups (Attard et al. 2015). The authors suggest that the opportunities for support, learning and communication are what make aphasia groups potent (Attard et al. 2015). This, and other fidelity monitoring of social support groups (Marshall et al. 2020), informed the development of the conversation groups fidelity checklist in VESFA.

TF activities in this research trial aimed to answer the following three research questions:

1. Was the treatment delivered as planned?
2. Which components most influenced treatment adherence scores?
3. How reliable were the fidelity checklists?

## 2 Methods

### 2.1 Design

The VESFA trial was a feasibility randomised controlled trial with two parallel arms as follows: VESFA and usual care (VESFA + UC) and a usual care control (UCC).

Full details of the VESFA intervention are reported in the VESFA therapy manual (available at https://figshare.com/articles/online_resource/VESFA_Therapy_Manual/26015641?file=46991479). In brief, VESFA was an 8‐week intervention comprising two individual ESFA and two group conversation sessions each week. Treatment stimuli were four conversation topics with 30 nouns in each topic. In individual sessions, participants retrieved the nouns and identified related verbs, adjectives and life stories. Conversation groups recapped topic words using the game Articulate, gave space for personal stories to be shared and played topic word Bingo, where target words on a card were woven into a narrative (see Tables 2 and 3 for session activities).

### 2.2 Fidelity Strategies

TF was supported in VESFA by strategies in four of the five fidelity areas identified by Bellg et al. (2004), see Table 1. One fidelity strategy, training providers, was minimally applicable as there was only one provider in the VESFA study (N.D.), a qualified Speech and Language Therapist (SLT) who had developed the intervention.

**TABLE 1:** Fidelity areas and strategies used in the VESFA trial.

Fidelity area	Strategies used in the VESFA trial
Study design	Pre‐specified participant inclusion/exclusion criteria Therapy manual specified 40h of intervention for all participants in the treatment arm Testing protocol specified 2h per testing session, with the same assessments to be delivered in the same order for both study arms. Planned to record sessions (1 individual session per participant per week and all groups) for later adherence checking
Training providers	Treatment was delivered by the research therapist who developed the intervention and wrote the manual. They are senior specialist SLT (BSc Hons in speech and language therapy and post‐registration MSc modules in acquired language impairments and research methods). A manualised protocol was followed.
Delivery of treatment	Treatment delivery followed the treatment protocol outlined in the VESFA Therapy Manual. Adverse events were recorded. Session videos were rated for adherence to the protocol.
Treatment receipt	Provider was an experienced aphasia therapist, with skills in providing hierarchical cues, supporting conversations (from Kagan 1998), and providing specific feedback. Inclusion criteria ensured those receiving the treatment could participate (had a minimum level of comprehension) and had room to improve (anomia identified at screening). A rationale was given for the ESFA activity when it was introduced. Feedback in the group was aimed to highlight how the use of strategies supported conversations. Practiced target words in naming, phrases and conversations in sessions
Treatment enactment	Provided challenge tasks. Participants were asked to identify a real‐world situation where the conversation could be practiced before the next group. Qualitative post‐therapy questionnaires specifically asked: ‘have you used the words and phrases practiced in EVA Park in real world conversations?’

### 2.3 Development of the Treatment Delivery Fidelity Checklists

Two fidelity checklists were developed to monitor treatment delivery. Following current recommendations (Walton et al. 2020), the fidelity checklists were based on the core activities identified during intervention development (Devane et al. 2025) and were informed by published TF checklists for ESFA (Kladouchou et al. 2017) and a TF checklist for a virtual group intervention (Marshall et al. 2020). The checklists were developed after the VESFA trial using materials (the therapy manual) that were specified before the trial began. The fidelity checklists were drafted by two authors (N.D. and S.M.) and reviewed and finalised through an iterative process with all authors. The core components of the treatment outlined in the checklists were verified by a workshop with the trial advisory group, which was comprised of four advisors with aphasia. In this workshop, the advisory group members generated what important activities drive change in therapies and identified what activities in VESFA were the important ones. The advisory group ratified all checklist items with the exception of two, rationale and feedback. Opinion was divided about whether it was necessary to provide a rationale for an activity. Additionally, advisory group members were very cautious about recommending verbal feedback. They felt it was so reliant on the skill of the therapist to be sensitive, that there was a chance that feedback might be detrimental to participant confidence. The rationale and feedback items remained in the checklist, but the sensitivity of feedback should be addressed in any future training of treatment providers. Fidelity Checklist A (Table 2) outlined the core components of the individual ESFA sessions. Fidelity Checklist B (Table 3) outlined the core components of the group conversation sessions.

**TABLE 2:** Fidelity Checklist A for the VESFA individual sessions.

(A) VESFA individual session					
Session ID:		Rater ID:			Date:
Item		Component	Please tick		Comments
			Done	Not done	
At the start	1	SLT gives rationale for chart activity.	□	□	
	2	SLT gives the opportunity to recap the items not named in the previous session.	□	□	
For each target			Done	Done	
			>/=75%	<75%	
	3	SLT provides a naming opportunity for the target word.	□	□	
	4	If the word cannot be named, the SLT follows a cueing hierarchy.	□	□	
	5	SLT elicits a minimum of 4/6 SFA chart categories.	□	□	
	6	SLT writes generated features.	□	□	
	7	SLT provides the opportunity for the participant to produce a phrase or sentence.	□	□	
			Done	Not done	
At the end	8	SLT asks the participant to name all the targets worked on within the topic.	□	□	
	9	SLT provides specific feedback.	□	□	
		*Examples: Number of words correct, effective strategies, supportive cues*.			
Total:			/9		

**TABLE 3:** Fidelity Checklist B for VESFA group sessions.

(B) VESFA conversation group					
Session ID:		Rater ID:			Date:
Item		Component	Please tick		Comments
			Done	Not done	
At the start (10 min)	1	SLT acknowledges each person in the group.	□	□	
	2	SLT provides the opportunity for participants to share news.	□	□	
	3	SLT introduces the group structure and topic of the session.	□	□	
Activity 1:	4	SLT describes ‘Articulate’:	□	□	
Articulate (20 min)		Includes the need to describe /give clues and guess.			
	5	In the vocabulary recap, SLT provides the opportunity for participants to retrieve target words.	□	□	
	6	SLT offers each participant a turn describing a target item.	□	□	
	7	SLT provides specific feedback on ‘articulate’ descriptions to participants.	□	□	
Activity 2:	8	SLT introduces the group's conversation topic.	□	□	
Conversation (30 min)					
	9	The virtual setting is linked to the topic.	□	□	
		*Example: Recipes are shared in the kitchen*.			
	10	SLT provides the opportunity for each participant to contribute to the conversation	□	□	
		*Example: Invites a contribution from someone who has not yet spoken*.			
	11	SLT provides specific feedback on the strengths of the conversation contributions.	□	□	
		*Examples: Range of words, structure of story*.			
Activity 3:	12	SLT describes the BINGO game.	□	□	
Bingo (15 min)					
		Includes the need to say the words on the BINGO card.			
	13	SLT provides the opportunity for participants to have a turn playing BINGO	□	□	
At the end (5–10 min)	14	SLT provides the opportunity for participants to reflect on their strengths, by asking	□	□	
		‘what have you been pleased to notice?’			
	15	SLT provides a challenge task or homework to be carried out independently before the next session.	□	□	
Overall	16	There are more than three demonstrations of enjoyment of the activity.	□	□	
		*Examples: Laughing, jokes*.			
Total:			/16		

### 2.4 Data Sampling

The VESFA intervention was an 8‐week intervention where participants (*n* = 16) received 4 sessions a week, in total ×16 individual ESFA sessions (S01–S16) and ×16 group sessions (grp01–grp16). Participants were treated in sets of three participants at a time, thus, ×6 (VESFA1–VESFA6) 8‐week sets were delivered between December 2020 and February 2022. A total of 192 sessions were planned in the VESFA trial (total sessions: 6 × 16 × 2 = 192). Twenty percent of the sessions were checked (20% of 192 = 38.4). A sample of 39 videos was randomly selected from the videoed sessions for fidelity rating. A list randomiser (www.random.org) was used to randomise the list of session videos. The first 20 individual sessions and 19 group sessions were selected from the list. The group and individual sessions were selected separately to ensure that both session types were well represented. The sample included a range of early and late sessions, see Appendix 1. All six sets were represented in the group sessions, see Appendix 1. Of these 39 videos, 35 were used for intra‐rater and inter‐rater reliability checks.

### 2.5 Data Allocation

Seven raters carried out the ratings of TF. They are identified here as Raters A–G. Rater A was a qualified SLT, Raters C–G were SLTs in training and Rater B was an undergraduate student in human communication. All raters were independent of the treatment study but familiar with the treatment to different degrees. Raters A and B watched videos of sessions to familiarise themselves with the treatment. Raters C–G had delivered the VESFA treatment in a student placement, independent of the VESFA trial, so had direct experience of the intervention.

Raters A and B were allocated 17 videos to rate and Raters C–G 15 videos to rate. This included videos that were watched twice for intra‐rater reliability, see Appendix 1.

### 2.6 Training Procedure

All raters attended a 1.5h training session. The training aims were for raters to understand the concept of TF, to be familiar with all items on the checklist and to gain experience in rating a range of items on the checklist. Following the training session, raters rated an example individual and example group session and then met to share ratings, discuss discrepancies and agree on additional rating criteria. These example videos were not used in the actual scoring. Disagreements were identified in decisions about the cueing hierarchy, feedback and rationales. Following these disagreements, further criteria were developed to guide raters. These were (1) if cueing was used, it needed to be hierarchical (started minimal and became more supportive) to be marked as ‘done’ (present); (2) feedback can only be considered ‘done’ if it is specific, for example, mentions something about what the participant did. For example, saying ‘good’ or ‘excellent’ does not represent specific feedback and (3) rationale needed to be specific to SFA. The SLT needed to refer to improving word finding/word retrieval and/or strengthening networks.

### 2.7 Procedure for Rating Adherence

Raters watched the videos of individual and group sessions with the relevant checklist and ticked ‘done’ or ‘not done’ adding notes where necessary. Raters were instructed to rate the videos in a private space and watch the full session with the checklist in front of them. For items where scores were above or below a certain percentage (>/ = 75%), raters were advised to keep a tally of each episode seen and calculate the score at the end of the video. In individual sessions, each video had a maximum score of 9. Group sessions in Set 1 had a maximum score of 14, and group sessions in Sets 2–6 had a maximum score of 16. Two components (Bingo game items) were added to the group protocol after a treatment review at the end of Set 1. Scores were either 1, for present or >/ = 75%, or 0, for not present or <75%, see checklists in Tables 2 and 3.

### 2.8 Procedure for Reliability

The scores of 35 session videos were compared between the same rater across different time points (intra‐rater) and two raters (inter‐rater) to determine the reliability of the checklists, Appendix 1 shows how these were allocated. A minimum of 10 days was left between ratings for intra‐rater reliability (Streiner et al. 2015). This aimed to ensure that the new ratings were based on what was seen in the video and not remembering the previous score.

### 2.9 Data Analysis

Adherence scores were calculated as a percentage: Present items were divided by total items and multiplied by 100. An adherence percentage of 80% or more represented high fidelity (Heilemann et al. 2014). Compliance was calculated as the percentage of sessions attended compared to sessions in the protocol. Intra‐ and inter‐rater reliability was calculated using a Cohens Kappa coefficient in the software Statistical Package for Social Sciences (SPSS, IBM, version 27). A kappa value can range from −1 to +1, see Table 4 (McHugh 2012).

**TABLE 4:** Interpretation of Cohen's kappa (McHugh 2012, 7).

Kappa value	Level of agreement	Percentage of the data that are reliable
0–0.20	None	0%–4%
0.21–0.39	Minimal	4%–15%
0.40–0.59	Weak	15%–35%
0.60–0.79	Moderate	35%–63%
0.80–0.90	Strong	64%–81%
Above 0.90	Almost perfect	82%–100%

## 3 Results

### 3.1 Study Design

Thirty four participants met inclusion criteria and equal numbers (*n* = 17) were randomised to the VESFA treatment arm and the Usual Care Control. 16/17 participants completed the 8‐week treatment. Ninety‐four percent of sessions ran as planned, with only 30/512 sessions cancelled. Seventy‐five percent of participants (12/16 participants) received over 90% (>36/40h) of the intended dose.

### 3.2 Delivery of Treatment

Of the 39 videos randomly selected for rating, 36 were rated (18%, 35/192). Three videos were not rated due to technical problems with the recordings, for example, no sound on the video. Despite this problem, the number of rated videos still met the recommended figure, between 10% and 20% (Behn et al. 2023; Hinckley and Douglas 2013).

#### 3.2.1 Adherence to Individual Session Protocol

Individual sessions had nine core components. Adherence scores ranged from 44% (4/9) to 89% (8/9) adherence, with an average of 78% (7/9). See Table 5 for scores for individual sessions.

**TABLE 5:** Adherence scores for individual sessions.

	Individual session	Components delivered and components planned	Adherence
		(actual score/maximum score)	$\frac{\mathrm{Actual}\mathrm{score}}{\mathrm{Maximum}\mathrm{score}}\times 100$
1	Ppt042.S11	7/9	78%
2	Ppt094.S07	7/9	78%
3	Ppt015.S14	6/9	67%
4	Ppt060.S02	6/9	67%
5	Ppt115.S07	7/9	78%
6	Ppt107.S14	8/9	89%
7	Ppt107.S12	8/9	89%
8	Ppt060.S08	8/9	89%
9	Ppt098.S08	7/9	78%
10	Ppt098.S09	7/9	78%
11	ppt113.S08	8/9	89%
12	ppt107.S15	8/9	89%
13	ppt053.S09	6/9	67%
14	ppt107.S06	8/9	89%
15	ppt087.S07	6/9	67%
16	ppt021.S15	4/9	44%
17	ppt021.S01	8/9	89%
18	ppt115.S12	7/9	78%
	Total	126/162	
	Mean	7/9	78%

Ppt = participant, S = session, for example, ppt042.S11 = participant 42, session 11.

#### 3.2.2 Adherence to Group Session Protocol

Group sessions had 16 core components. Two components were added to the review after Set 1, therefore all Set 1 groups (VESFA1) had a total of 14 core components. Adherence scores ranged from 50% (7/14) to 100% (16/16), with an average of 84% (13–16). See Table 6 for adherence scores for group sessions.

**TABLE 6:** Adherence scores for group sessions.

	Group session	Components delivered and components planned	Adherence
		(actual score/maximum score)	$\frac{\mathrm{Maximum}\mathrm{score}}{\mathrm{Actual}\mathrm{score}}\times 100$
1	VESFA5.Group01	13/16	81%
2	VESFA1.Group05	7/14	50%
3	VESFA6.Group10	14/16	88%
4	VESFA5.Group04	12/16	75%
5	VESFA6.Group03	14/16	88%
6	VESFA4.Group05	16/16	100%
7	VESFA4.Group14	14/16	88%
8	VESFA5.Group03	13/16	81%
9	VESFA2.Group05	15/16	94%
10	VESFA1.Group10	11/14	79%
11	VESFA4.Group07	15/16	94%
12	VESFA6.Group08	15/16	94%
13	VESFA4.Group11	14/16	88%
14	VESFA1.Group08	11/14	79%
15	VESFA3.Group14	15/16	94%
16	VESFA4.Group09	15/16	94%
17	VESFA1.Group06	9/14	64%
18	VESFA3.Group10	12/16	75%
	Total	235/282	
	Mean	13/16	84%

*Note*: VESFA# refers to the number of the set. There were 6 sets and 16 groups in each set.

#### 3.2.3 Overall Adherence

Overall adherence was calculated by adding the adherence scores of individual and group sessions together ((126 + 235)/(162 + 282) = 81.3). It was done to give a single summary score for the whole intervention delivery. The overall adherence to the protocol in VESFA intervention was 81%.

#### 3.2.4 Ratings by Component

**FIGURE 1:**
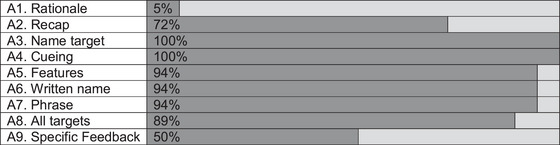
Visualisation of the frequency that each checklist item was rated present (dark grey) or not present (light grey) in the individual sessions.

To understand what components of the sessions were driving variability in the adherence ratings we looked at the item responses. Figure 1 shows the frequency which items were present in the individual sessions. Two items scored low for adherence, (A1) rationale for the activity was only seen once in 18 sessions and specific feedback (A9) was seen 50% (9/18) of the time. All other components were present more than 72% of the time. The naming of the target word (A3) and the use of hierarchical cueing by the therapist (A4) were present in all rated sessions.

If we remove Item A1 (rationale), the adherence rating rises to an average of 88% across the individual sessions and the overall adherence to 86%.

**FIGURE 2:**
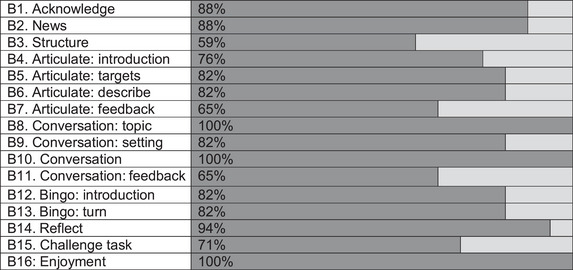
Visualisation of the frequency that each checklist item was rated present (dark grey) or not present (light grey) in the group sessions.

The frequency of components present in the group sessions is shown in Figure 2. In the group sessions, a description of the group structure (B3) was the least present component, seen in 59% of rated sessions (10/17). Specific feedback (B7, B11) was seen in 65% of the sessions rated (11/17). The introduction of the conversation topic, the opportunity for participants to take a turn in the conversation and evidence of enjoyment (B8, B10, B16) were seen in all group sessions rated.

#### 3.2.5 Reliability of the Checklists

##### 3.2.5.1 Fidelity Checklist A

Fidelity Checklist A rated the individual sessions. Two raters independently rated 17 individual sessions, see Table 7. Inter‐rater reliability was perfect for 65% of sessions (11/17) with Kappa = 1, *p* < 0.001, moderate for 37% of sessions (4/17), Kappa = 0.63–0.67 and weak for 12% sessions (2/17), Kappa = 0.44 and 0.52.

**TABLE 7:** Results for inter‐ and intra‐rater reliability rating for Fidelity Checklist A.

Fidelity Checklist A. Individual sessions							
Inter‐rater reliability				Intra‐rater reliability			
Rater	Kappa	Sig.	95% CI	Rater	Kappa	Sig.	95% CI
A & B	1	<0.001	1–1	A	1	<0.001	1–1
A & B	0.63	<0.001	0.31–0.95	A	1	<0.001	1–1
A & B	0.67	<0.006	0.22–1.12	A	1	<0.001	1–1
A & B	0.52	<0.002	0.18–0.85	A	0.70	<0.001	0.42–0.98
A & B	0.63	<0.001	0.31–0.95	A	1	<0.001	1–1
E & D	1	<0.001	1–1	E	1	<0.001	1–1
E & D	1	<0.001	1–1	E	1	<0.001	1–1
E & D	1	<0.001	1–1	E	1	<0.001	1–1
E & D	1	<0.001	1–1	E	1	<0.001	1–1
E & D	1	<0.001	1–1	E	1	<0.001	1–1
C & G	1	<0.001	1–1	C	1	<0.001	1–1
C & G	0.44	0.048	−0.2–1.09	C	1	<0.001	1–1
C & G	1	<0.001	1–1	C	1	<0.001	1–1
C & G	1	<0.001	1–1	C	1	<0.001	1–1
C & G	1	<0.001	1–1	C	1	<0.001	1–1
D & C	0.62	<0.003	0.32–0.91	D	1	<0.001	1–1
D & C	1	<0.001	1–1	D	1	<0.001	1–1
Mean:	0.85			Mean	0.98		

Abbreviations: CI, confidence interval; Sig., significance.

The 17 sessions were also rated by the same rater twice, Table 7. Intra‐rater reliability was perfect for 94% of sessions (16/17). For the one session with disagreement, reliability was moderate with Kappa = 0.70, *p* < 0.001.

##### 3.2.5.2 Fidelity Checklist B

Fidelity Checklist B rated the group sessions. Two raters independently rated 15 group sessions, see Table 8. Inter‐rater reliability was perfect for 20% of the sessions (3/15), moderate for 40% of sessions (6/15), weak for 27% (4/15) and minimal for 13% (2/15).

**TABLE 8:** Results for inter‐ and intra‐rater reliability rating for Fidelity Checklist B.

Fidelity Checklist B. Group sessions							
Inter‐rater reliability				Intra‐rater reliability			
Rater	Kappa	Sig.	95% CI	Rater	Kappa	Sig.	95% CI
B & A	0.54	<0.001	0.20–88	B	0.72	<0.001	0.45–0.99
B & A	1	<0.001	1–1	B	1	<0.001	1.0–1.0
B & A	1	<0.001	1–1	B	1	<0.001	1–1
B & A	0.62	<0.001	0.32–0.92	B	1	<0.001	1–1
B & A	0.72	<0.001	0.45–0.99	B	0.72	<0.001	0.45–0.99
F & E	0.39	<0.001	0.03–0.74	F	0.32	<0.001	−0.03–0.67
F & E	0.72	<0.001	0.45–0.99	F	0.72	<0.001	0.45–0.99
F & E	0.72	<0.001	0.45–0.99	F	0.54	<0.001	0.20–0.88
F & E	0.65	<0.001	1–1	F	0.29	0.034	−0.12–0.70
G & F	0.54	<0.001	0.20–0.88	G	1	<0.001	1–1
G & F	0.54	<0.001	0.20–0.88	G	1	<0.001	1–1
G & F	1	<0.001	1–1	G	1	<0.001	1–1
G & F	0.78	<0.001	0.47–1.08	G	1	<0.001	1–1
G & F	0.29	<0.034	−0.12–0.70	D	1	<0.001	1–1
G & F	0.54	<0.001	0.20–0.88	D	0.72	<0.001	1–1
Mean:	0.67			Mean:	0.80		

Abbreviations: CI, confidence interval; Sig., significance.

The 15 sessions were also rated by the same person twice, Table 8. Intra‐rater reliability was perfect for 60% of sessions (9/15), moderate for 20% of sessions (3/15), weak for 7% of sessions (1/15) and minimal for 13% of sessions (2/15).

### 3.3 Treatment Receipt

To support the receipt of treatment, all participants had a minimum level of comprehension (screening criterion >/ = 6/10 on FAST comprehension). This ensured that they could access the virtual world, EVA Park and the focus on verbal language in VESFA.

Four components of the checklists supported treatment receipt: rationale for activities (Item A1), cuing (Item A4), specific feedback on actions (Item A9) and conversation practice (Item B8). A rationale for the activity and feedback on actions were the least present in the VESFA therapy sessions rated (see Figure 1). However, the opportunity to practice the use of the target words (Item B8, Figure 2) in naming, phrases and conversations was present in all sessions rated, which meant participants used the target words during therapy sessions and in subsequent therapy sessions.

### 3.4 Treatment Enactment

Treatment enactment was supported through the provision of challenge tasks. Challenge tasks were presented to participants in 71% of the sessions rated. Additionally, questions in the post‐therapy questionnaire probed treatment enactment through the following question: ‘have you used the words and phrases practiced in EVA Park in real world conversations?’. Ninety‐four percent of participants (14/15) answered positively to this question. Examples include how the practice in EVA Park supported talking to a waiter in a restaurant ‘a meal—me in a restaurant, sentences.’ (ppt21) and speaking about holidays in an EVA Park session inspired a conversation about holidays in the real word.

## 4 Discussion

In the VESFA trial, TF strategies covered study design, treatment delivery, treatment receipt and treatment enactment. Study design strategies ensured 94% of sessions ran as planned and 75% of participants (12/16) received over 90% (>36/40h) of the intended dose. The average TF across all sessions rated was 81%, demonstrating a high degree of fidelity in the delivery of the VESFA intervention (Heilemann et al. 2014). The mean TF for individual sessions was 78% and 84% for group sessions. In terms of treatment receipt strategies, a rationale for the activities and specific feedback for performance were not practised as intended by the therapist. Nevertheless, participants consistently practised target words both in individual sessions and in conversations in the group sessions, demonstrating treatment receipt. Ninety‐four percent of participants (14/15) reported the words and phrases practiced in EVA Park in real‐world conversations, indicating treatment enactment.

### 4.1 Delivering Treatment as Planned

Adherence TF of 81% is comparable to other EVA Park interventions, with the scripts therapy reporting over 80% (Marshall et al. 2023) and the social support intervention reporting 81.9% (Marshall et al. 2020). However, adherence was lower than face‐to‐face ESFA interventions (Kladouchou et al. 2017) and other aphasia interventions (Bacon et al. 2021; Heilemann et al. 2014) where TF was reported at over 90%. Although the average TF score was strong, one session scored less than 50% adherence (ppt021.S15, Table 5). This was the individual session 15/16. In Sessions 13–15, the therapy protocol dictates that the therapist recaps all 30 words in the three topics across the three sessions (see VESFA Therapy Manual). Thus, the SFA chart is not completed for each word, as it was earlier in the intervention. It is likely that Items A5–A7 (the features, the written name and the word in a phrase) were not elicited in this session leading to the low score. A future trial could address how to check these different session types within the VESFA intervention.

### 4.2 Components That Most Influenced Treatment Adherence

A closer look at the ratings shows that item A1.Rationale was only seen once in session 021.S01. This was the only Session 1 in the sample. It is likely that the activity rationale was given when the activity was first introduced but not in subsequent sessions. This raises the following question: Is providing a rationale for the activity in every session a core component of the VESFA therapy? Understanding why treatment works is a construct within the acceptability of an intervention (Sekhon et al. 2017). ‘Intervention coherence’, the participant's understanding of the intervention and how it works, and ‘perceived effectiveness’, the extent to which the intervention is perceived to work, contributes to acceptability, impacting adherence and therefore clinical outcomes. Adult learning literature outlines that a rationale, understanding why we need to learn something, will support intrinsic motivation to the task (Gom 2009). We know from clinical experience that information needs to be given more than once to be taken on, and this is confirmed in the literature (Kessels 2003). This suggests that participants would need to hear the rationale more than once to understand why an activity is done.

The other component with low ratings was feedback (A9.Specific Feedback, B7.Articulate feedback and B11.Conversation feedback). This was marked as present if the feedback provided specific information related to the task. For example, ‘telling us the location helped us to guess the word’ would be rated as present but ‘well done’ would be rated as not present. It has long been established that feedback can improve performance (Thorndike 1927), and feedback remains a key strategy in adult learning (Ahmad et al. 2022). In aphasia rehabilitation, feedback is multi‐functional (Simmons‐Mackie et al. 1999). It serves to shape target behaviours, encourage, boost confidence, maintain a partnership, set a tempo in the task, consolidate the therapist–client roles and communicate rules (Simmons‐Mackie et al. 1999). This checklist only captured the feedback that shaped the target behaviours. Physiotherapists give more motivational than informational feedback during stroke rehabilitation activities (Stanton et al. 2015). It is likely that this is the case with SLTs too. Motivational feedback can highlight a sense of success and boost confidence leading to increased self‐efficacy. Future studies could check for both forms of feedback.

It is interesting to note that the two items that were least present in the sessions, rationale and feedback, were the two that the advisory group suggested might not be core activities, and those most difficult to rate consistently during training. These items support the recipient of the treatment to understand why the treatment works and what they can do to benefit from treatment. There are interventions where the repetitive practice is the driver of change, for example, those that aim to re‐organise neural networks. Motivational feedback may support and enhance such practice. In behavioural interventions, understanding why you should change your behaviour may also influence outcomes, for example, when is the right moment to use the strategy that you have worked on. Here providing rationales and specific feedback, for example, referencing the task and effective strategies, is useful. Literature on feedback in aphasia therapy highlights that feedback has multiple functions in therapy, including encouraging and boosting confidence as well as modifying or maintaining target behaviours (Simmons‐Mackie et al. 1999). VESFA pulls on both these mechanisms to strengthen the semantic neural networks and use the features’ descriptions to compensate for word‐finding occurrences in conversations (see Devane et al. 2025). As such they are core components of the intervention. Adherence testing is carried out to pick up the issues with treatment delivery. Here it has revealed issues with the consistency of rationale and feedback. The VESFA therapy manual should be updated to place emphasis on a regular description of the task rationale and examples of specific task‐related feedback, and the timing of feedback. Future TF studies could give more opportunities for benchmarking in training.

### 4.3 Reliability of Rating

Moving to the reliability of the checklists, when both checklists are conflated, inter‐rater reliability was moderate (average Kappa of 0.76) and intra‐rater reliability was strong (average Kappa of 0.89) showing that the fidelity checklists were reliable. The mean inter‐rater reliability for the individual session checklist was strong, but moderate for the group session checklist. A similar pattern was seen in intra‐rater reliability where the reliability of the individual session checklist was almost perfect and strong for the group session checklist. Interestingly, the TF scores were higher for group than individual sessions, but the reliability of the scoring of the group checklist was poorer. Low intra‐rater reliability scores suggest a training issue. Some items on the fidelity checklist were objective, for example, participant produces the target word, and some were more subjective, for example, feedback was specific to the task. The latter example can be harder to judge. Raters had one opportunity to discuss differences in judgements in the training. They may have benefited from more opportunities to benchmark, particularly for feedback and cueing hierarchy items. Disagreements by item were predominantly for judgements about feedback in both individual and group sessions. This matches a comparable aphasia fidelity paper where disagreements were due to judgements about differential feedback (Bacon et al. 2021).

The range in reliability scoring may have been influenced by the number of raters. Seven research students rated the session videos. They had a wide range of experience; one rater had no experience of rehabilitation, five were first‐year SLTs in training and one was a qualified SLT. These differences would have contributed variability to the ratings. This is evident in the range of reliability ratings. The ratings for the group sessions were less reliable. It is possible that the components in the groups required more judgement, for example, what constitutes specific feedback versus generic feedback.

### 4.4 Treatment Receipt

Treatment receipt strategies are often carried out in the screening of a trial, that is, screening criteria exist that ensure participants have the potential to benefit from the intervention, and occasionally during a trial. An example of a treatment receipt strategy during a trial is the use of a checklist to ensure that the participant was attentive, understood, and attempted to do the behavioural task of interest (Spell et al. 2020). The VESFA trial addressed treatment receipt in screening with a minimum comprehension criterion and a maximum naming ability criterion and during the trial with prompts to practice skills in everyday conversations (challenge tasks).

### 4.5 Treatment Enactment

Reporting a measurement of treatment enactment is rare in aphasia studies. In a recent review (Behn et al. 2023), two studies demonstrated treatment enactment (Breitenstein et al. 2017; Palmer et al. 2015), and two studies (Behn et al. 2021; Rose et al. 2022) partially met the review criteria. In the VESFA trial, treatment enactment was addressed by asking about enactment in an interview. This method was also used in a recent aphasia peer‐befriending trial (Hilari et al. 2021). One study aimed to capture the enactment of therapy skills by observing participants in conversation (Harrison 2019).

### 4.6 Limitations

Planning for TF before the intervention is delivered can increase the chance of high fidelity ratings (Behn et al. 2023; Brogan et al. 2019). Fidelity checklists can be referred to before or after each session to keep adherence high. This process would detect possible drift from the protocol that could be corrected in subsequent sessions. In this study, the fidelity checklists were not developed prior to the intervention. This may have contributed to the fact that some core components were not delivered consistently across sessions. Having a TF checklist from the start and completing a treatment provider self‐assessment after each session can keep the emphasis on the core components, especially in long delivery periods, such as in this study (18 months).

As indicated above, a limitation of this study was the retrospective development of the checklist. Prospective, iterative fidelity checking can pick up on issues and adjust them in a long trial. Moreover, more benchmarking in training, where raters practice rating a video and then discuss and resolve uncertainties in scoring, could have led to better reliability in the scoring.

A possible concern is that our selection of videos did not enable us to determine whether TF varied across the participants, as would have been the case if we evaluated 20% of sessions per participant. We opted to check a proportion of the total sample, not a proportion per participant for a number of reasons. First, all participants were treated by the same therapist, removing one potential source of variation. Second, our participant selection criteria aimed to minimise other variables, for example, in levels of comprehension. Third, checking 20% of sessions per participant would have substantially increased the number of videos required for rating, and raised the overall percentage to well above the recommended figure. Although our selection method was random, all participants were included in the chosen sessions. Individual session fidelity scores (see Table 5) suggest that there was just one outlier, with a low score of 44%. However, this does not seem to be a participant effect, as the same individual featured in a second‐rated video, which scored 89%.

This study did not explore the fidelity of assessment. Although guidance now exists for TF (Bellg et al. 2004), there is very little guidance for researchers on assessment fidelity (Richardson et al. 2016). In the VESFA trial, the assessments were completed by a number of testers. In future studies, assessment sessions could be videoed and rated against a checklist and/or tester self‐report checklists.

### 4.7 Considerations for Future Research

Training of intervention providers was not an issue in this trial, but a future trial with multiple sites would need consistent training for providers. Here, fidelity of training might also be assessed.

## 5 Conclusion

A range of TF strategies was embedded within the trial protocol (study design, treatment delivery, treatment receipt and treatment enactment). VESFA demonstrated high adherence to the core components of the intervention, with 75% of participants receiving more than 90% of the intended dose. The reliability of the checklist was moderate to strong. The components that elicited the most drift were providing a rationale for activities and the provision of specific feedback. Developing the fidelity checklist in advance of the treatment delivery may mitigate this in future trials. Training for fidelity raters should include more opportunities to benchmark. The therapy manual should be updated to include a task rationale and the provision of specific task‐related feedback. Overall, this study adds to the evidence that aphasia therapy can be delivered faithfully in varying formats, including when hosted on a virtual reality platform. Monitoring and reporting TF is a valuable component of aphasia treatment research.

## Ethics Statement

Ethical approval was obtained from the Senate Research Ethics Committee at City University of London (ETH1920‐1223).

## Consent

All participants gave informed written consent.

## Conflicts of Interest

The authors declare no conflicts of interest.

## Supporting information



Supporting Information

## Data Availability

The data that support the findings of this study are available from the corresponding author, N.D., upon reasonable request.
