# Supplementary material for: Treatment Fidelity in a Feasibility Trial of the Aphasia Intervention, Virtual Elaborated Semantic Feature Analysis
Source: Int J Lang Commun Disord. 2025 May 23;60(3):e70054. doi: 10.1111/1460-6984.70054 (PMC12099486; doi:10.1111/1460-6984.70054)
Supplement: Supplementary file 1 — Supporting Information [file JLCD-60-0-s001.docx]

Appendix 1: Allocation of individual session and group session videos to raters A-G

| **Video:** | | **Rater:** | | |
| --- | --- | --- | --- | --- |
|  |  | **Adherence** | **Inter-rater reliability** | **Intra-rater reliability** |
| **Individual sessions** | ppt042.S11 | A | B | A |
|  | ppt094.S07 | A | B | A |
|  | ppt015.S14 | A | B | A |
|  | ppt060.S02 | A | B | A |
|  | ppt115.S07 | A | B | A |
|  | ppt087.S11 | E | D | E |
|  | ppt009.S12 | E | D | E |
|  | ppt007.S16 | E | D | E |
|  | ppt098.S08 | E | D | E |
|  | ppt098.S09 | E | D | E |
|  | ppt113.S08 | C | G | C |
|  | ppt107.S15 | C | G | C |
|  | ppt053.S09 | C | G | C |
|  | ppt107.S06 | C | G | C |
|  | ppt087.S07 | C | G | C |
|  | ppt065.S09 | D | C | D |
|  | ppt021.S15 | D | C | D |
|  | ppt075.S09 | D | C | D |
|  | ppt088.S13 | A |  |  |
|  | ppt115.S12 | B |  |  |
| **Group sessions** | VESFA5grp01 | B | A | B |
|  | VESFA1grp05 | B | A | B |
|  | VESFA6grp10 | B | A | B |
|  | VESFA5grp4 | B | A | B |
|  | VESFA6grp03 | B | A | B |
|  | VESFA5grp04 | F | E | F |
|  | VESFA4grp05 | F | E | F |
|  | VESFA4grp14 | F | E | F |
|  | VESFA5grp03 | F | E | F |
|  | VESFA2grp05 | F | E | F |
|  | VESFA1grp10 | G | F | G |
|  | VESFA4grp07 | G | F | G |
|  | VESFA6grp08 | G | F | G |
|  | VESFA4grp11 | G | F | G |
|  | VESFA1grp08 | G | F | G |
|  | VESFA3grp14 | D | C | D |
|  | VESFA4grp09 | D | C | D |
|  | VESFA1grp06 | A |  |  |
|  | VESFA3grp10 | B |  |  |

*ppt=participant, S=session e.g., ppt042.S11= participant 42, session 11.*

*VESFA1=set 1, grp=group e.g., VESFA5grp4= The fifth set of participants, group 4*
